# Supplementary material for: Circulating cell free DNA during definitive chemo-radiotherapy in non-small cell lung cancer patients – initial observations
Source: PLoS One. 2020 Apr 28;15(4):e0231884. doi: 10.1371/journal.pone.0231884 (PMC7188247; doi:10.1371/journal.pone.0231884)
Supplement: S1 Fig — ●: Tumor change from PET/CT scan (blood sample no 2) to fourth cfDNA blood sample (11th fraction). +: Tumor change from PET/CT scan (blood sample no 2) to fifth cfDNA blood sample (22nd fraction). cfDNA: circulating cell-free DNA. (PDF) [file pone.0231884.s001.pdf]

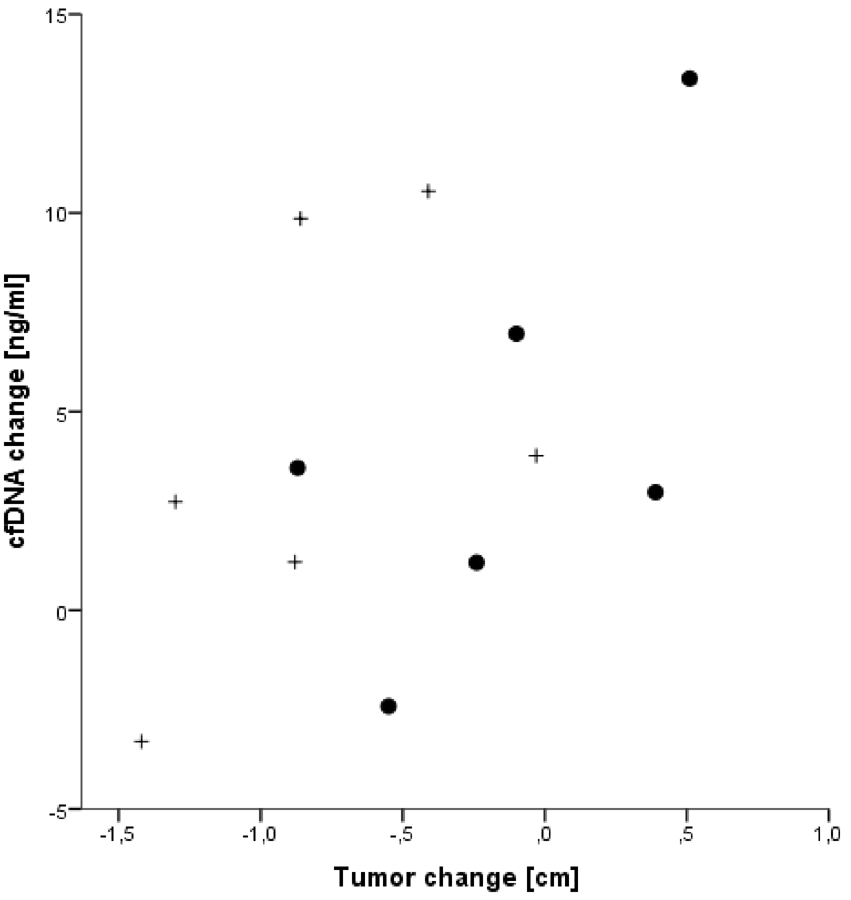


*Figure S1. Scatter plot of cfDNA change as a function of tumor shrinkage measured on CT. ●: Tumor change from PET/CT scan (blood sample no 2) to fourth cfDNA blood sample (11th fraction). +: Tumor change from PET/CT scan (blood sample no 2) to fifth cfDNA blood sample (22nd fraction). cfDNA: circulating cell-free DNA.*
