# Supplementary material for: Circulating cell free DNA during definitive chemo-radiotherapy in non-small cell lung cancer patients – initial observations
Source: PLoS One. 2020 Apr 28;15(4):e0231884. doi: 10.1371/journal.pone.0231884 (PMC7188247; doi:10.1371/journal.pone.0231884)

Figure S2. All sWGS reads following the in silico size selection data with tMAD scores in patients from part 2 of the study.

Patient 5 – Baseline 6,401,689 reads

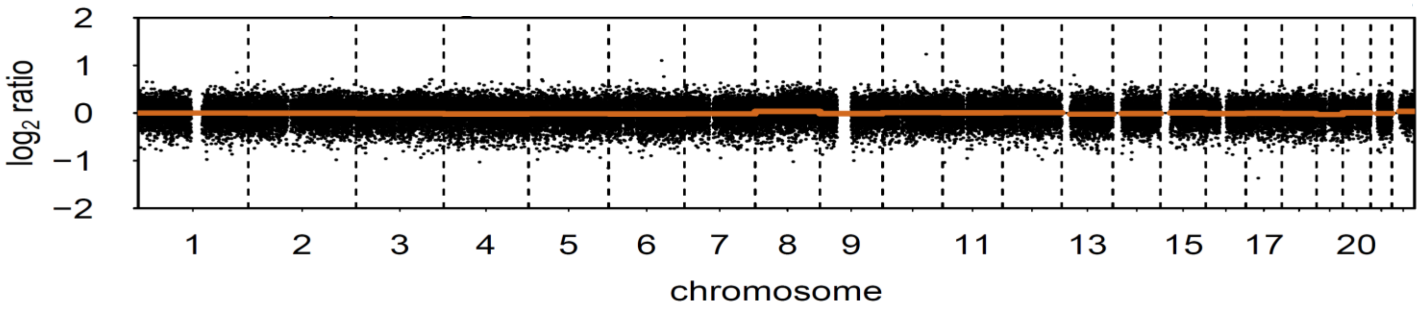

Patient 5 – PET/CT 7,103,971 reads

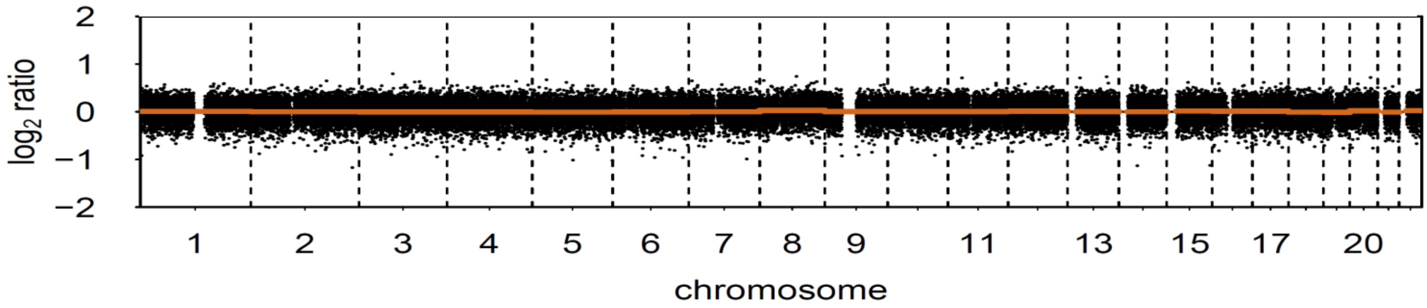

Patient 5 – 1st fraction 6,739,532 reads

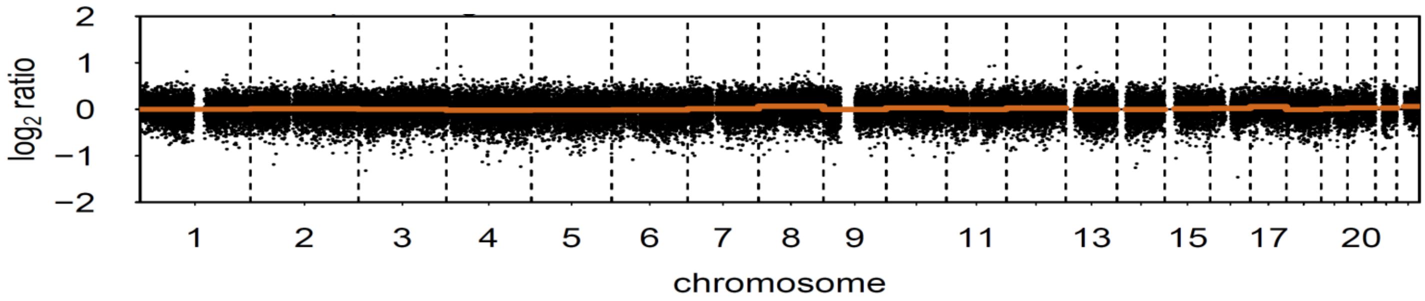

Patient 5 – 11th fraction 5,216,640 reads

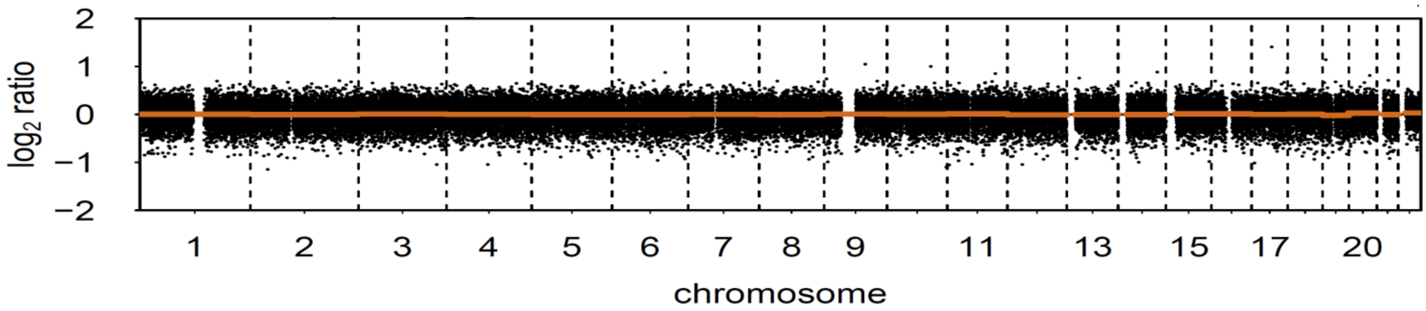

Patient 6 – Baseline 7,749,428 reads

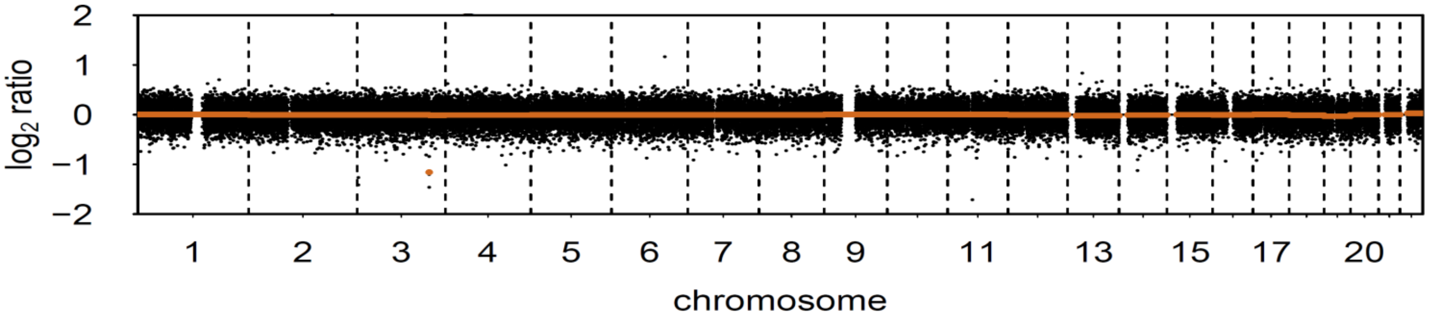

Patient 6 – PET/CT 11,027,373 reads

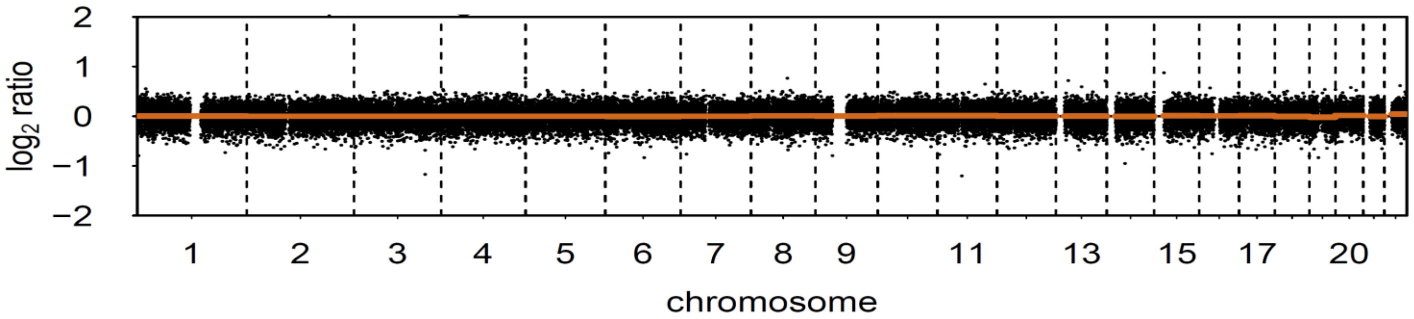

Patient 6 – 1st fraction 7,786,541 reads

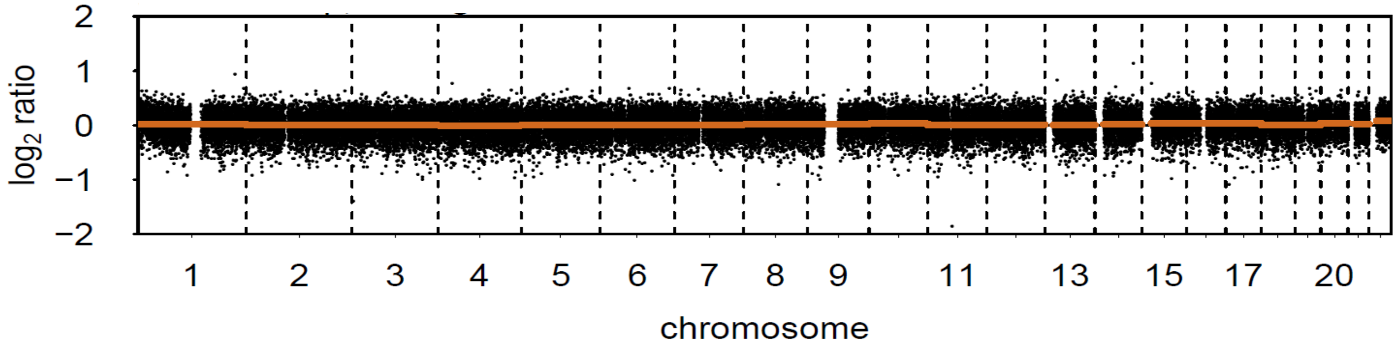

Patient 6 – 11th fraction 7,201,007 reads

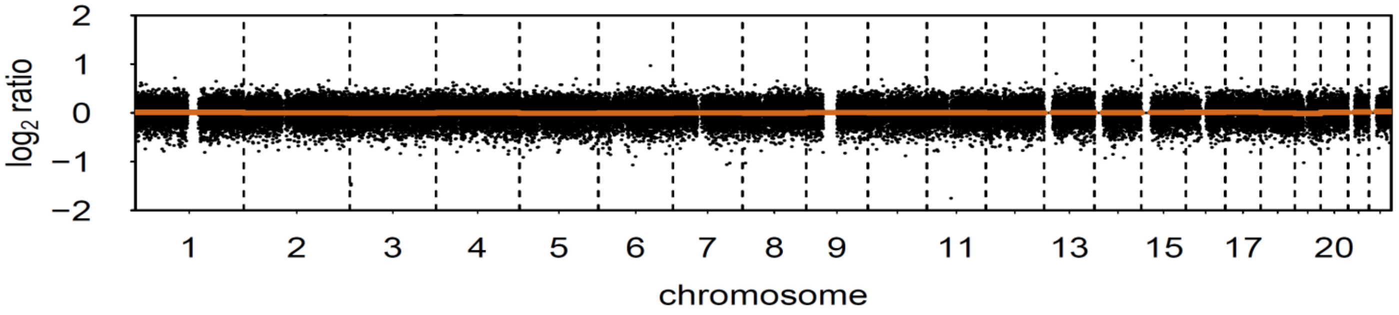

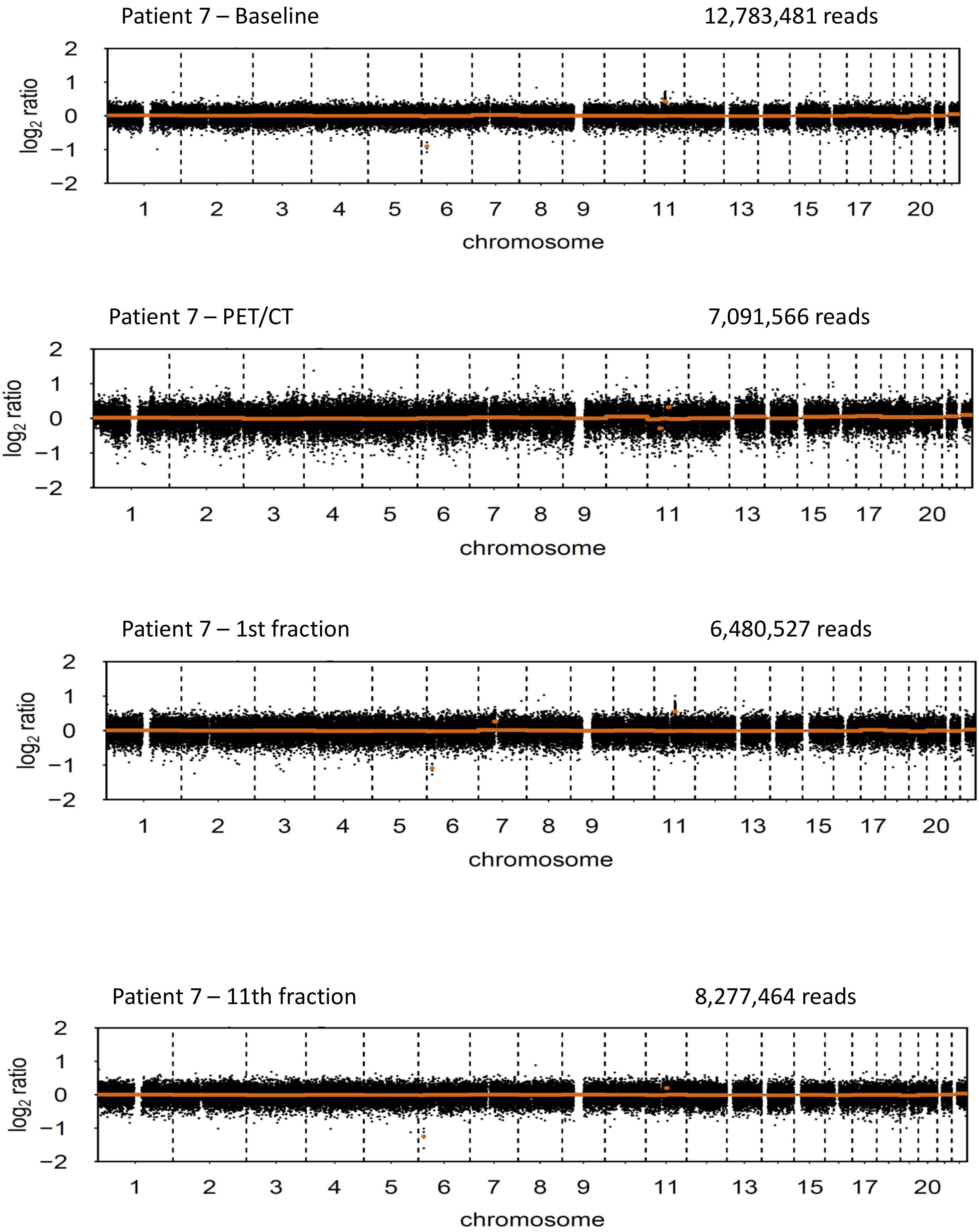

Patient 7 – Baseline
12,783,481 reads
Patient 7 – PET/CT
7,091,566 reads
Patient 7 – 1st fraction
6,480,527 reads
Patient 7 – 11th fraction
8,277,464 reads
log2 ratio
chromosome

Patient 8 – Baseline 5,299,819 reads

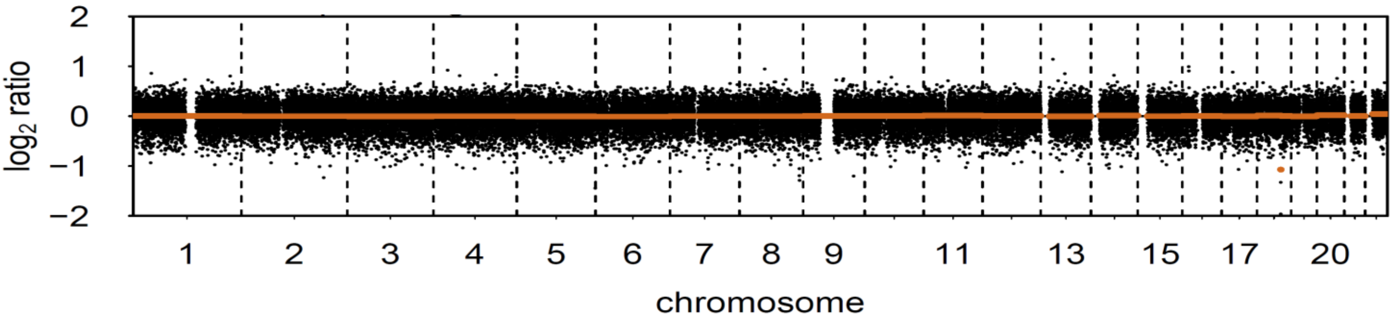

Patient 8 – PET/CT 3,974,443 reads

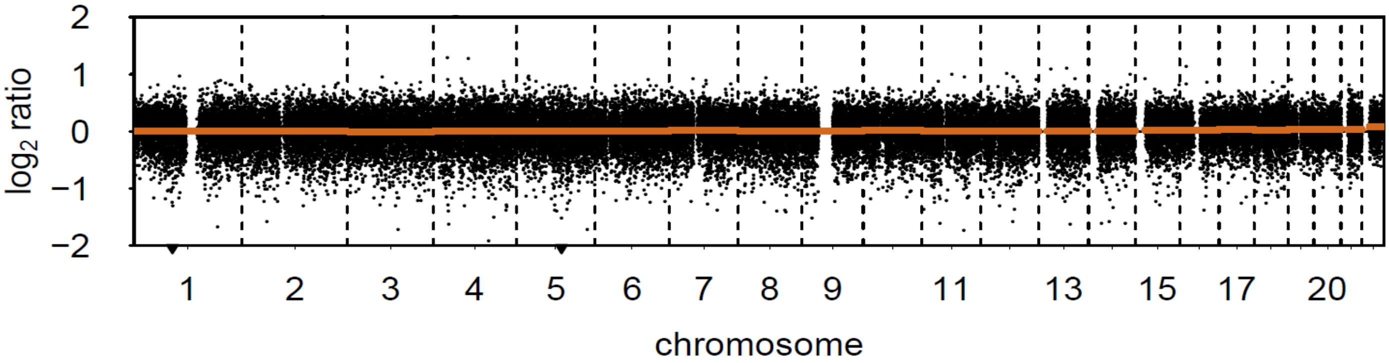

Patient 8 – 1st fraction 5,446,781 reads

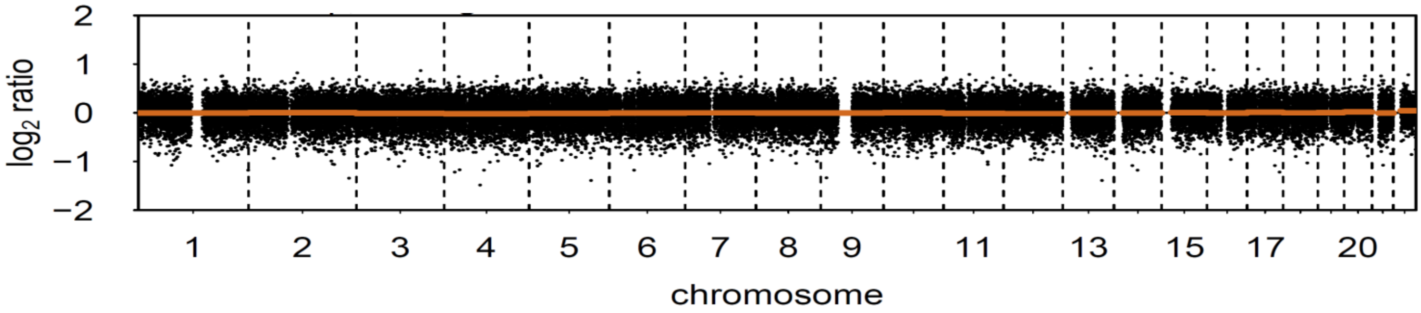

Patient 8 – 11th fraction 9,761,532 reads

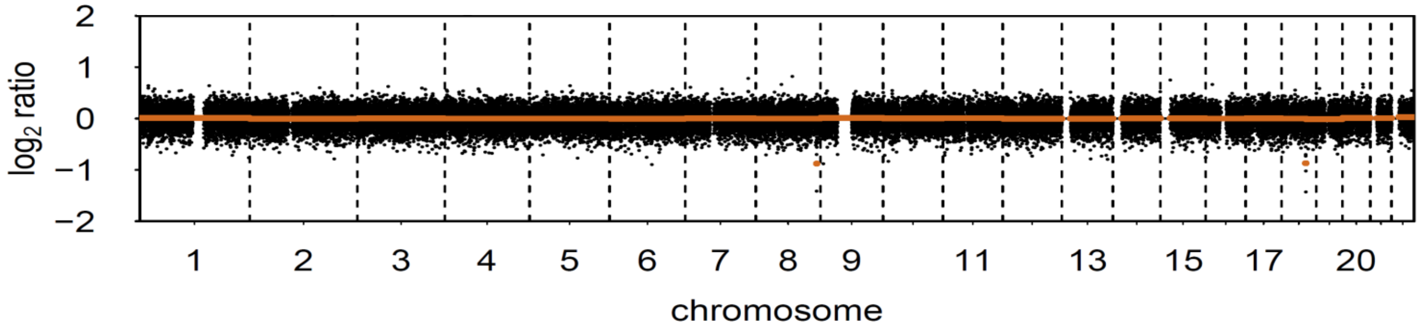

Patient 9 – Baseline 6,822,075 reads

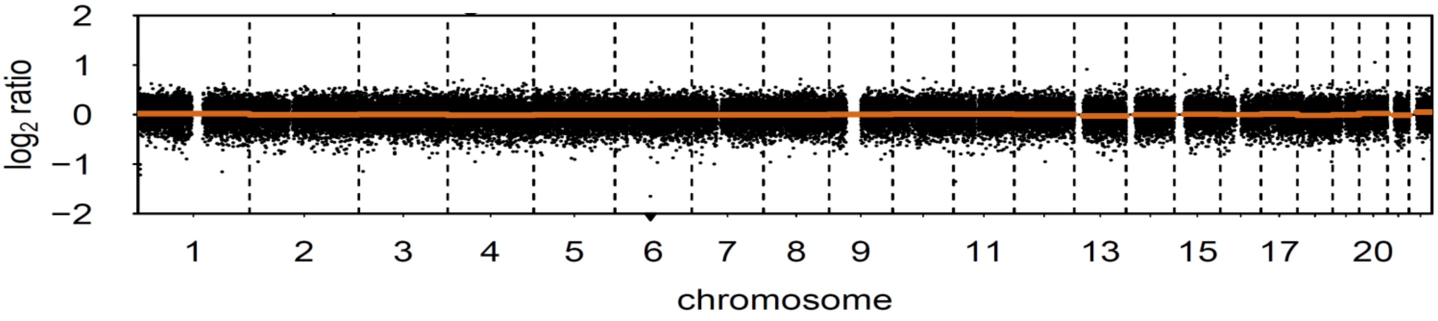

Patient 9 – 1st fraction 5,987,394 reads

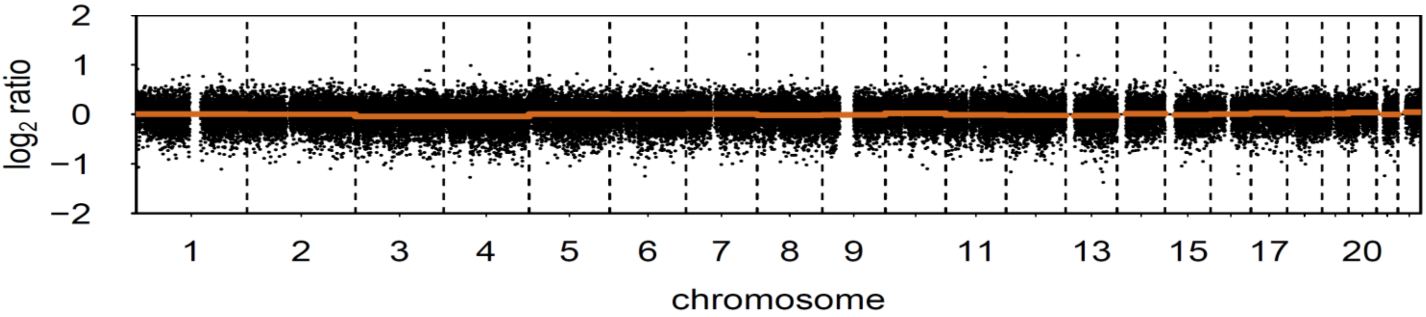

Patient 10 – Baseline 10,510,857 reads

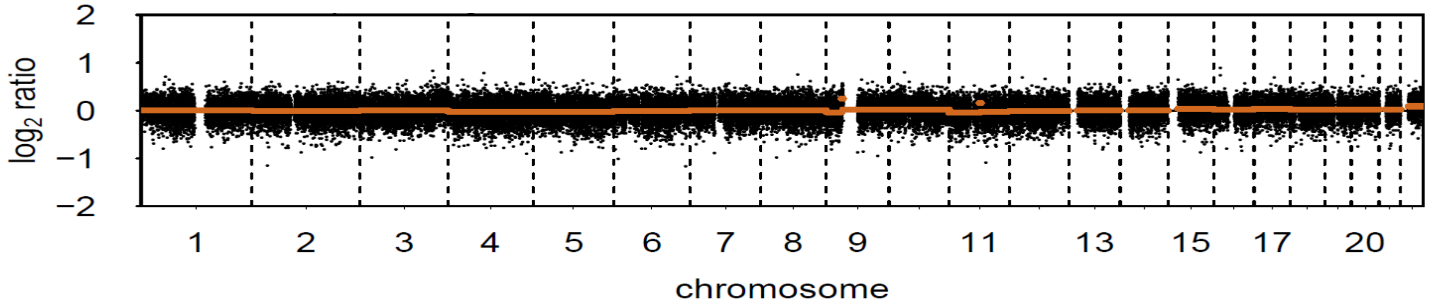

Patient 10 – PET/CT 7,410,195 reads

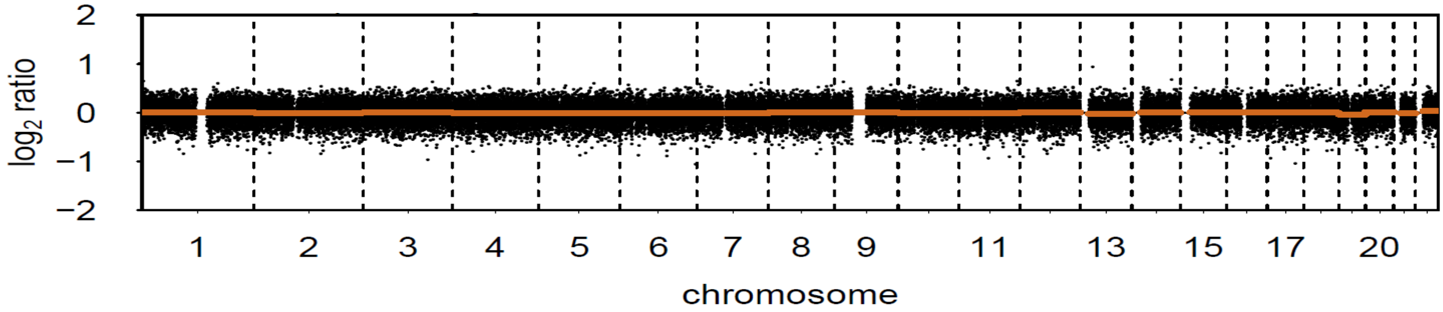

Patient 10 – 1st fraction 10,488,818 reads

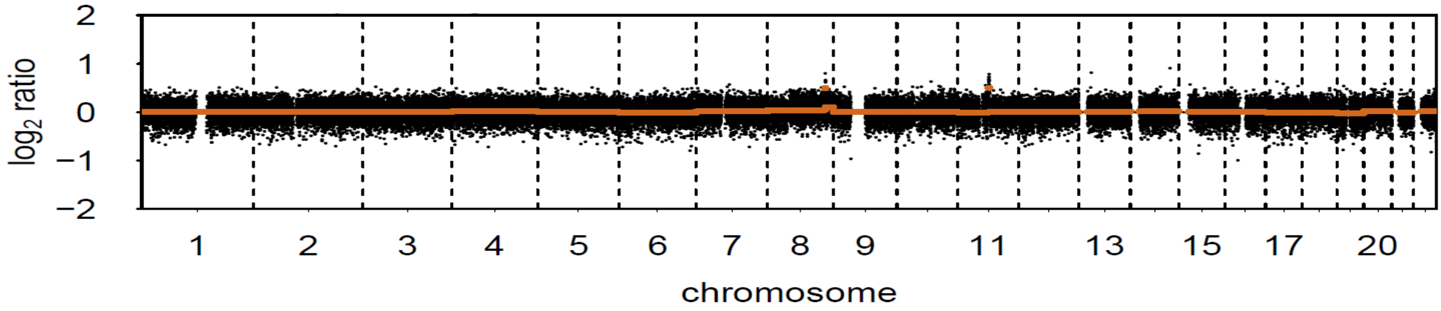

Patient 10 – 11th fraction 10,658,435 reads

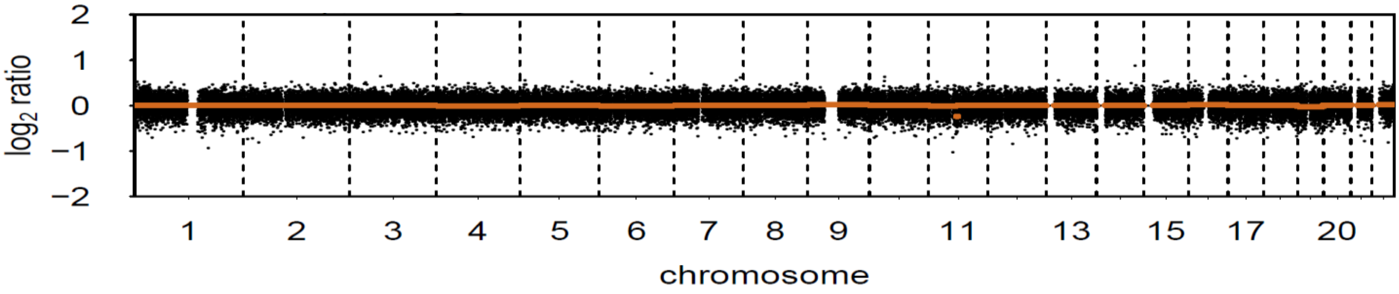

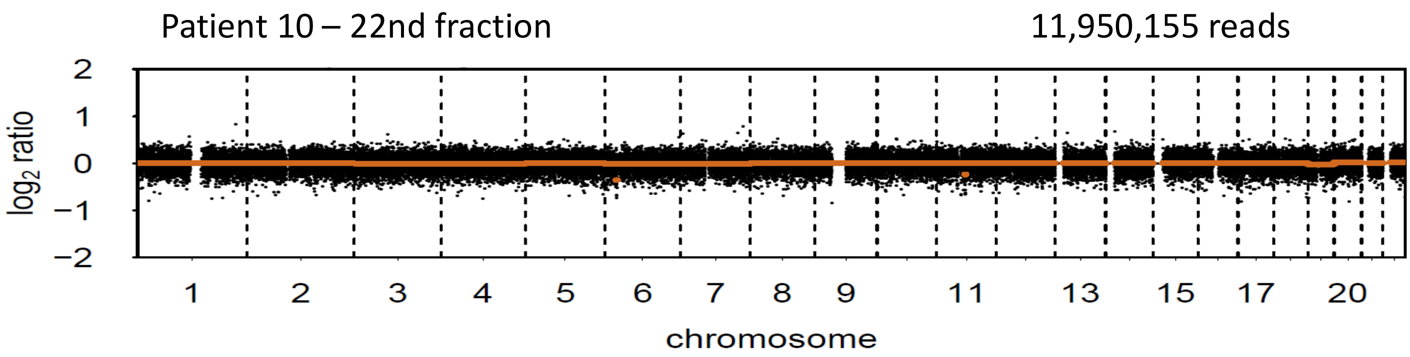

Patient 10 – 22nd fraction
11,950,155 reads
log2 ratio
2
1
0
−1
−2
1
2
3
4
5
6
7
8
9
11
13
15
17
20
chromosome

Patient05.01
SZ tMAD = 0.027 (detected)

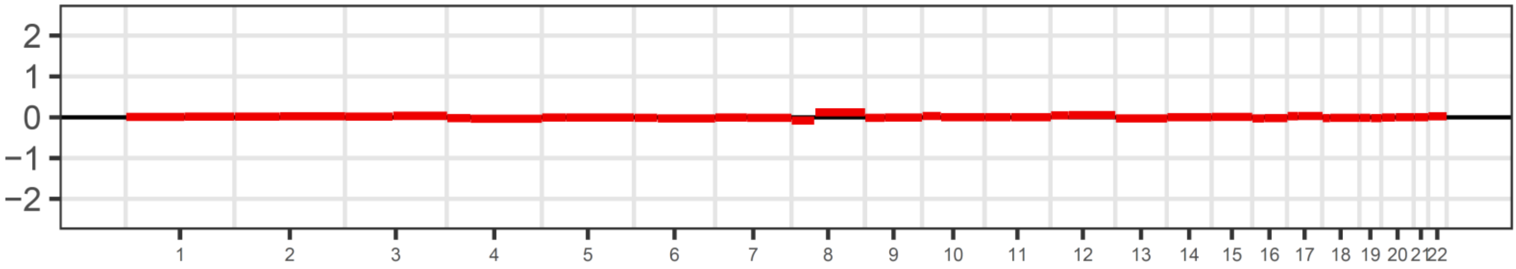

Patient05.02
SZ tMAD = 0.027 (detected)

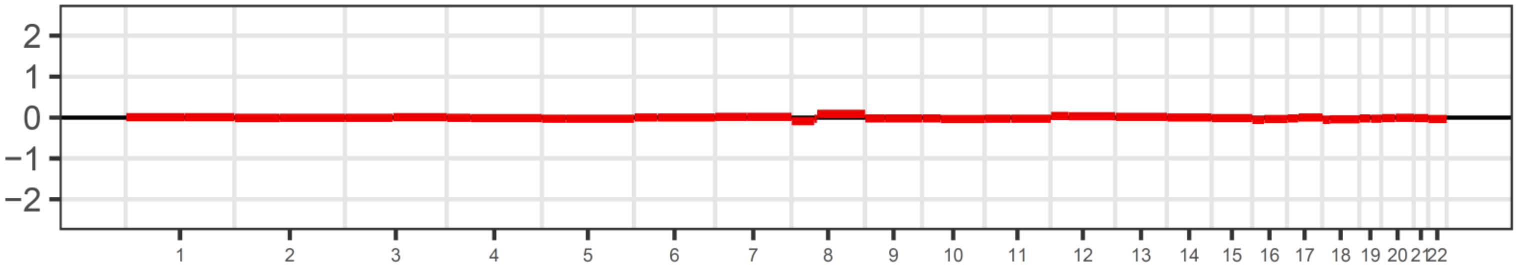

Patient05.03
SZ tMAD = 0.025 (detected)

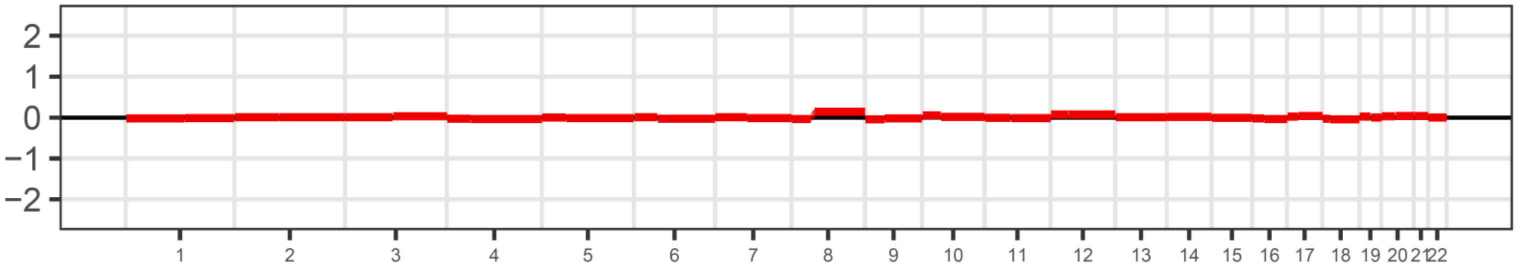

Patient05.04
SZ tMAD = 0.015 (detected)

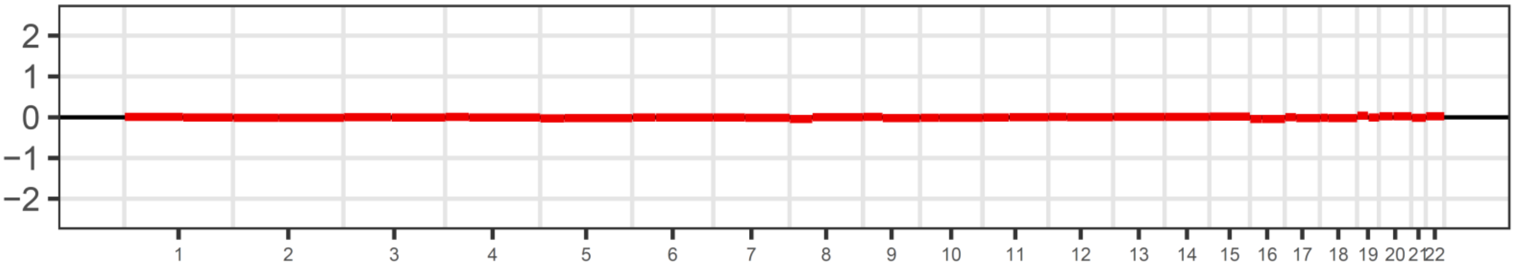

Patient06.01
SZ tMAD = 0.016 (detected)

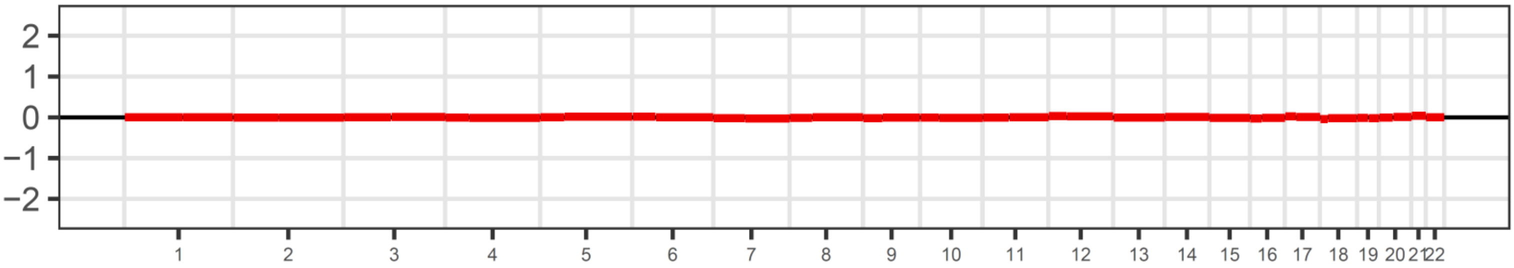

Patient06.02
SZ tMAD = 0.012 (undetected)

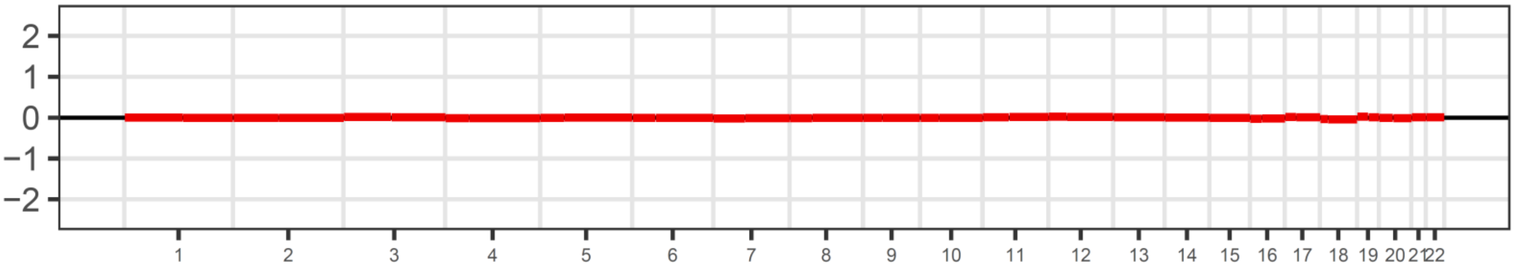

Patient06.03
SZ tMAD = 0.016 (detected)

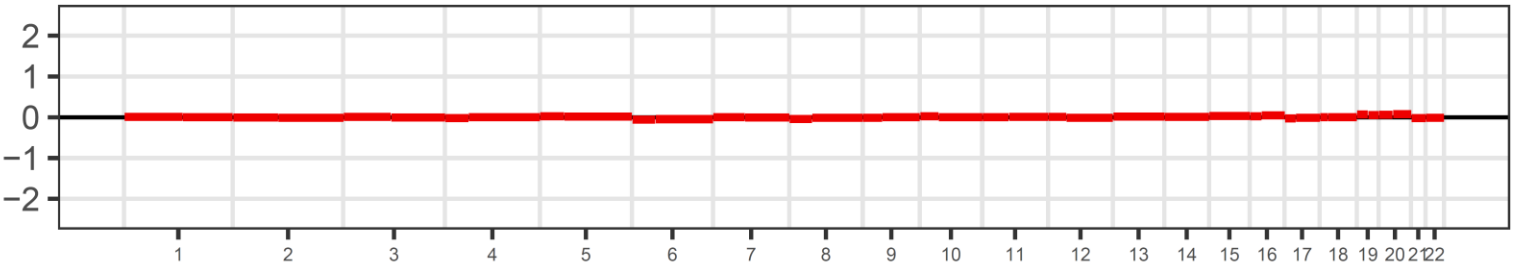

Patient06.04
SZ tMAD = 0.007 (undetected)

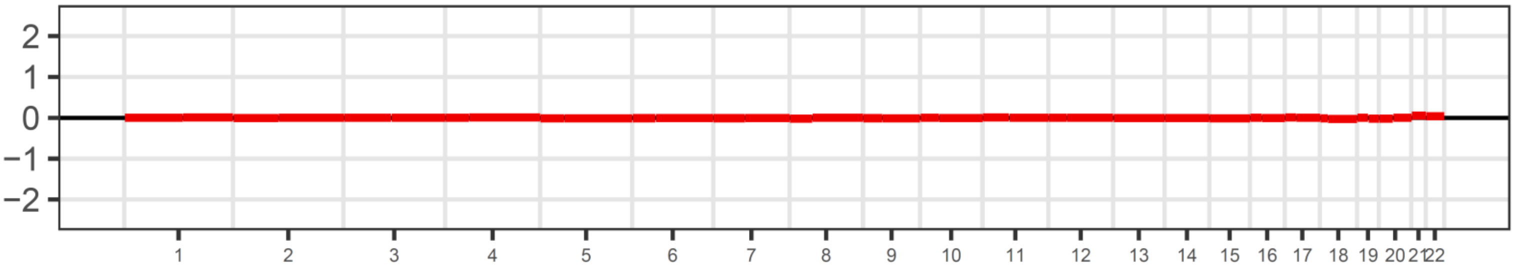

Patient07.01
SZ tMAD = 0.024 (detected)

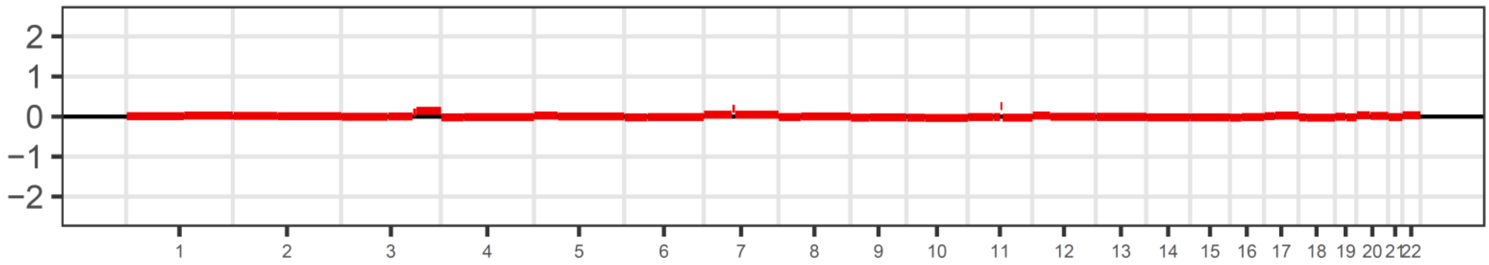

Patient07.02
SZ tMAD = 0.018 (detected)

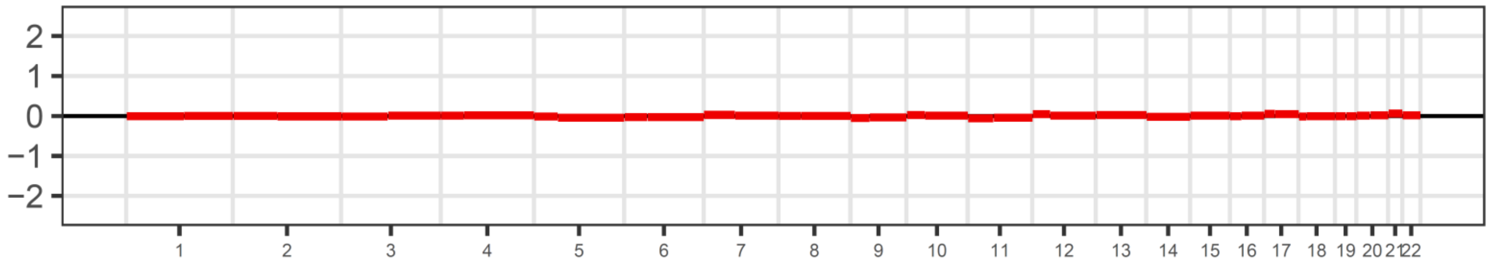

Patient07.03
SZ tMAD = 0.025 (detected)

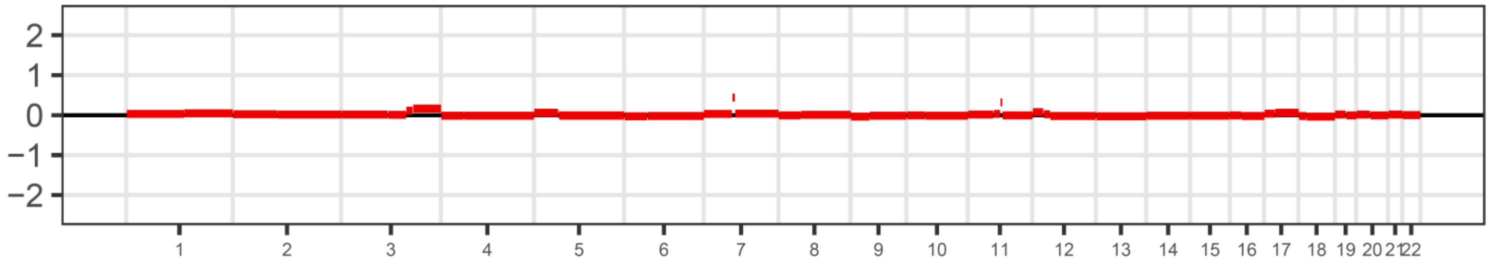

Patient07.04
SZ tMAD = 0.013 (undetected)

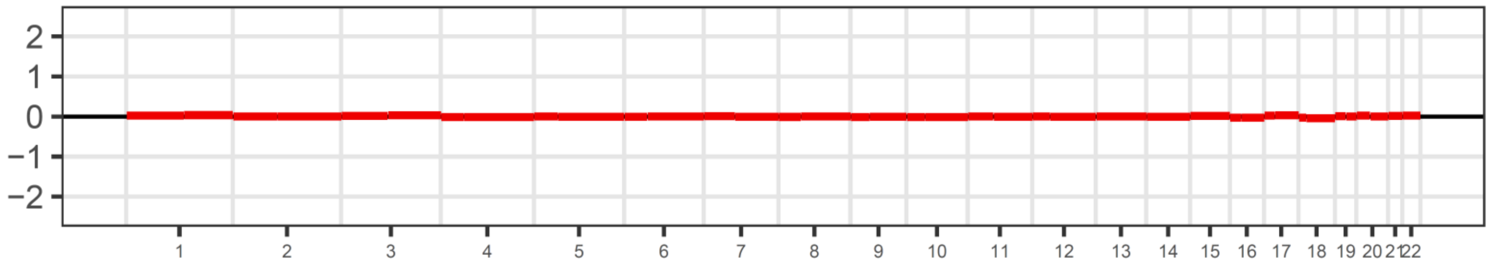

Patient08.01
SZ tMAD = 0.016 (detected)

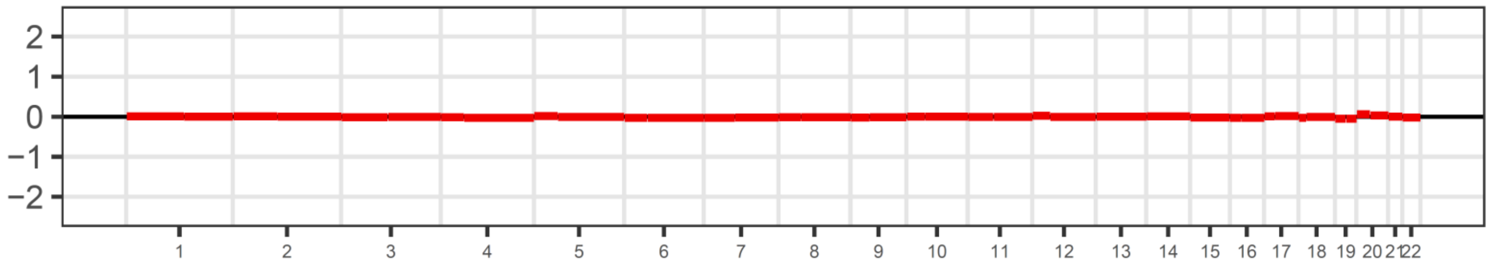

Patient08.02
SZ tMAD = 0.027 (detected)

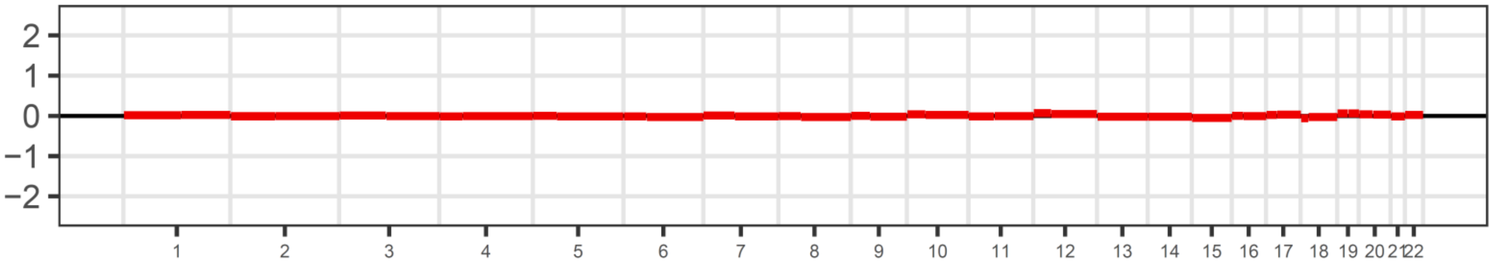

Patient08.03
SZ tMAD = 0.013 (undetected)

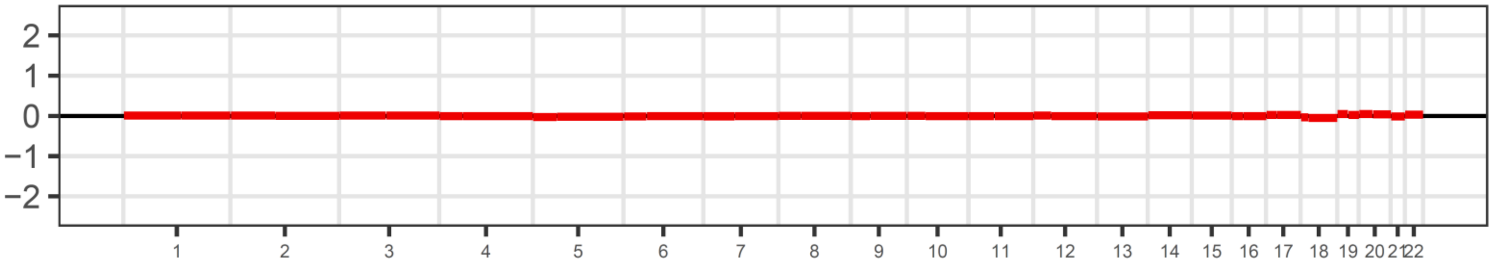

Patient08.04
SZ tMAD = 0.014 (undetected)

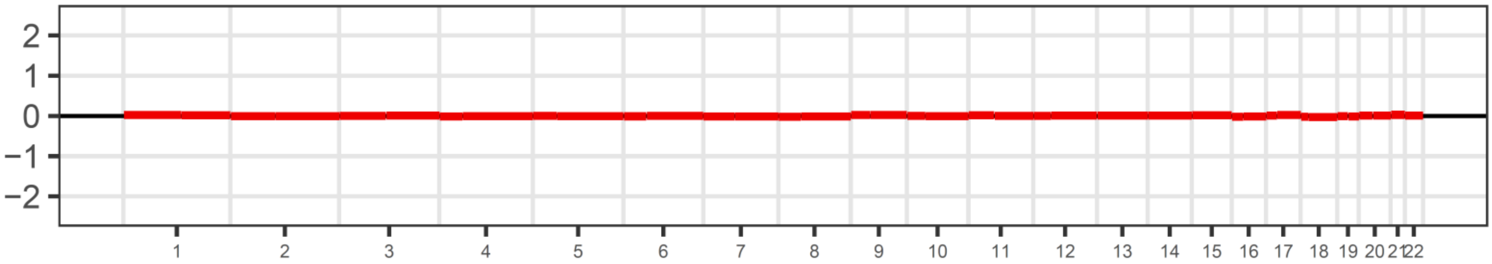

Patient09.01
SZ tMAD = 0.019 (detected)

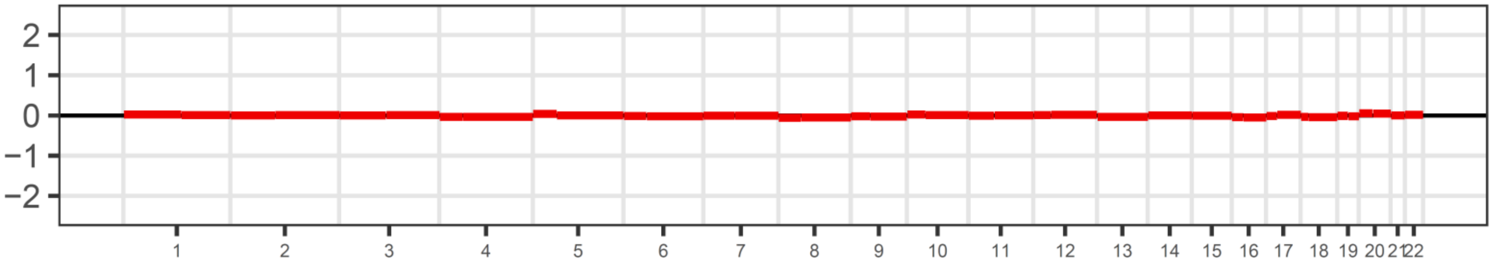

Patient09.03
SZ tMAD = 0.035 (detected)

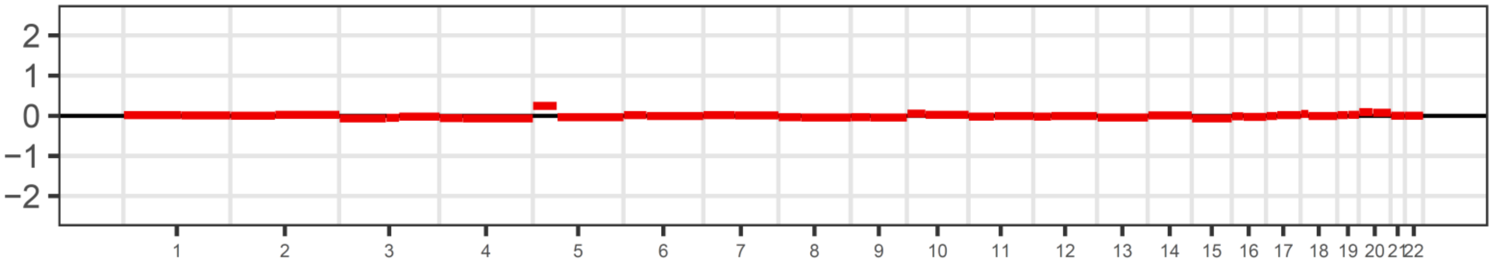

Patient10.01
SZ tMAD = 0.026 (detected)

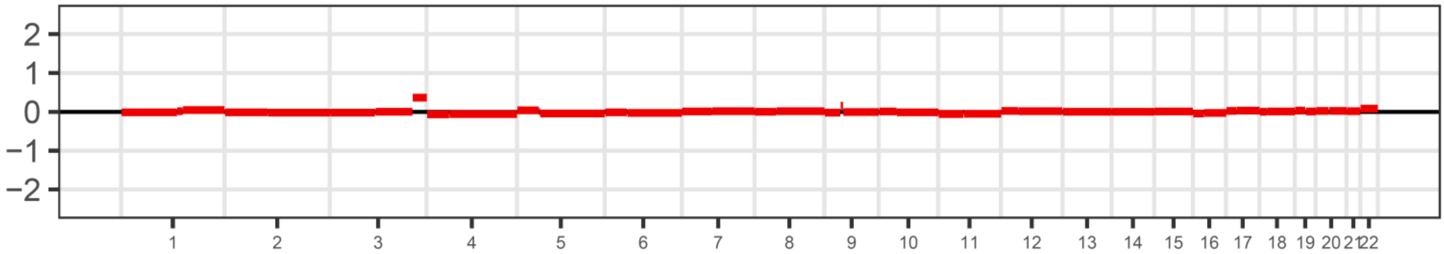

Patient10.02
SZ tMAD = 0.016 (detected)

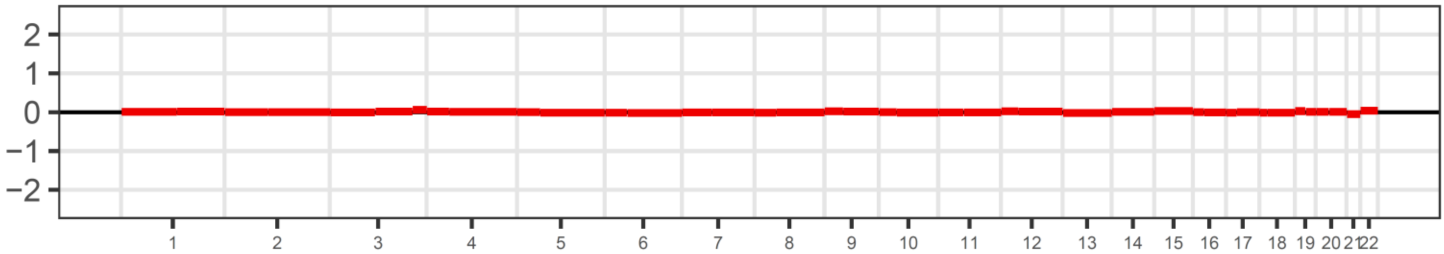

Patient10.03
SZ tMAD = 0.016 (detected)

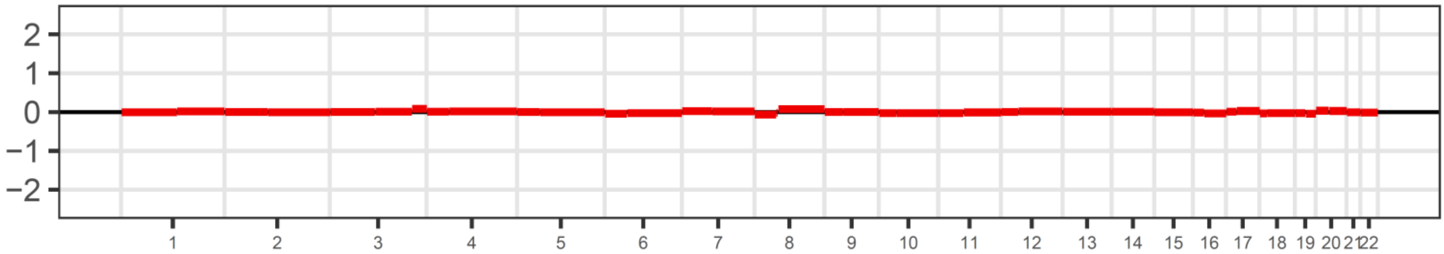

Patient10.04
SZ tMAD = 0.012 (undetected)

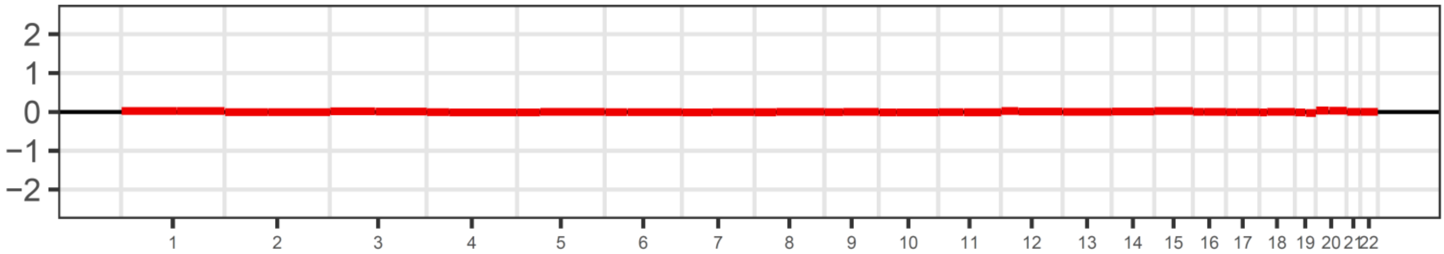

Patient10.05
SZ tMAD = 0.014 (undetected)

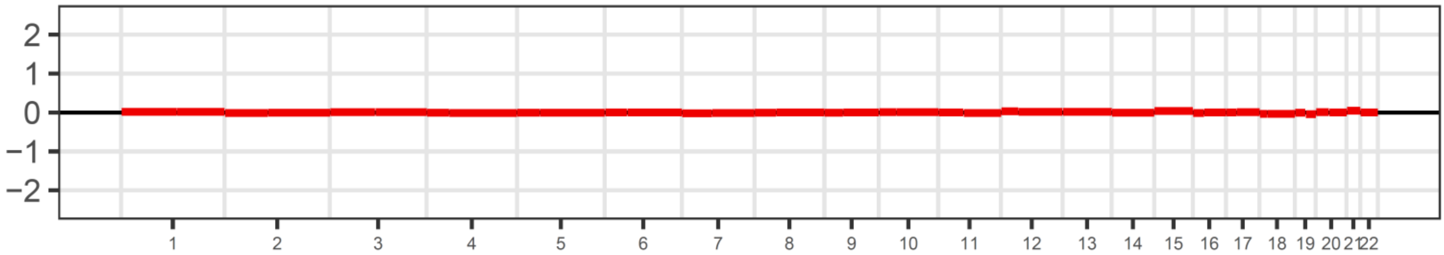

Supplement: S2 Fig — (PDF) [file pone.0231884.s002.pdf]
