# Supplementary material for: Circulating cell free DNA during definitive chemo-radiotherapy in non-small cell lung cancer patients – initial observations
Source: PLoS One. 2020 Apr 28;15(4):e0231884. doi: 10.1371/journal.pone.0231884 (PMC7188247; doi:10.1371/journal.pone.0231884)
Supplement: S3 Fig — Gy: Gray. cfDNA: circulating cell free DNA. PET/CT: positron emission tomography/computer tomography. (PDF) [file pone.0231884.s003.pdf]

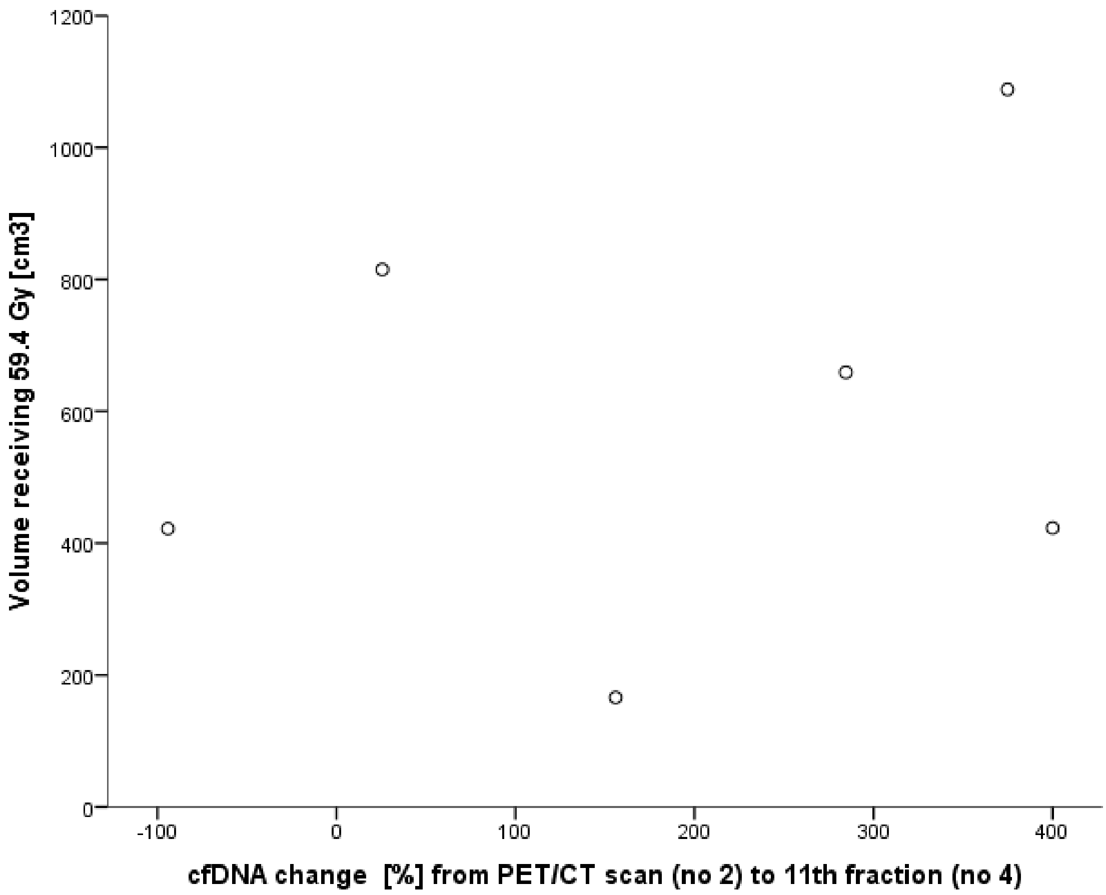

Figure S3. Scatter plot of Volume [cm3] of the body receiving 59.4 Gy (90% of the prescribed 66 Gy) and a function of cfDNA change. Gy: Gray. cfDNA: circulating cell free DNA. PET/CT: positron emission tomography/computer tomography.
