# Supplementary material for: Circulating cell free DNA during definitive chemo-radiotherapy in non-small cell lung cancer patients – initial observations
Source: PLoS One. 2020 Apr 28;15(4):e0231884. doi: 10.1371/journal.pone.0231884 (PMC7188247; doi:10.1371/journal.pone.0231884)
Supplement: S4 Fig — cfDNA: circulating cell free DNA. GTV: Gross tumor volume. PET/CT: positron emission tomography/computer tomography. SCC: Squamous cell carcinoma. AC: Adenocarcinoma. (PDF) [file pone.0231884.s004.pdf]

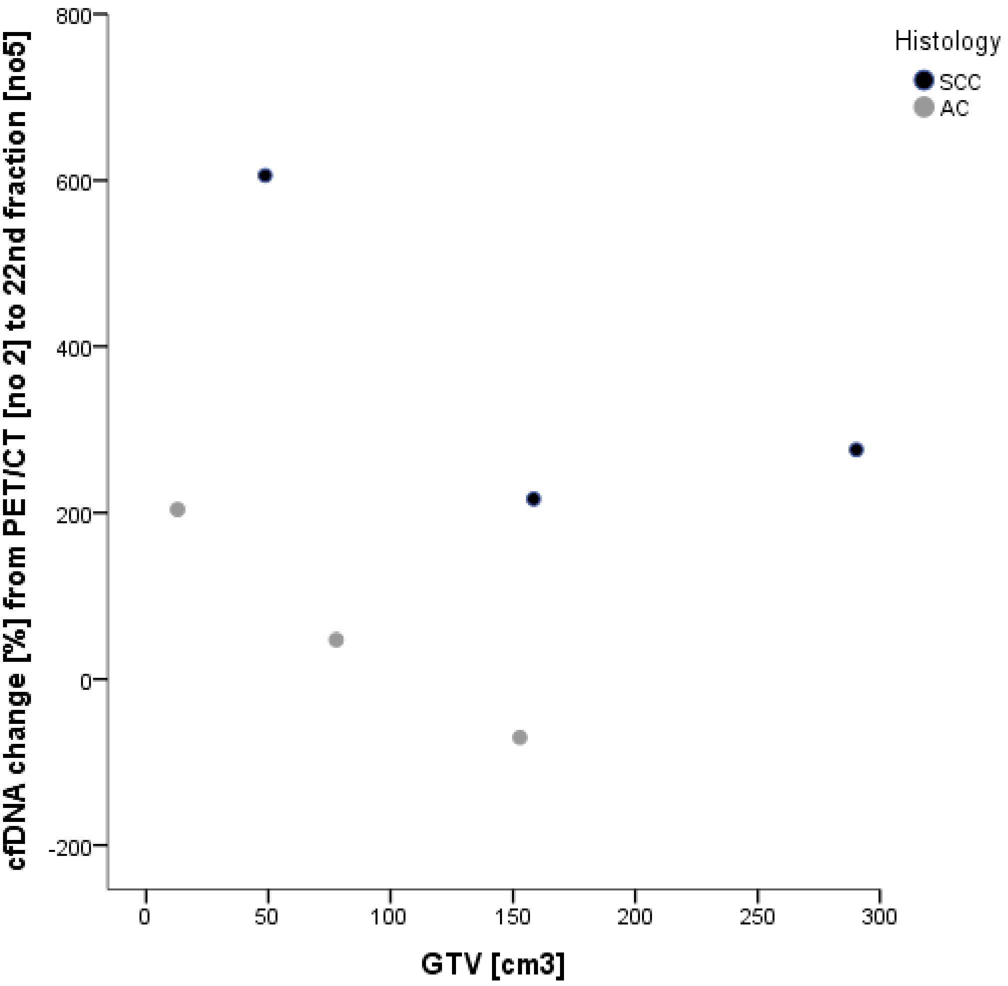

Figure S4. Scatter plot of cfDNA level change as a function of GTV divided by histology. cfDNA: circulating cell free DNA. GTV: Gross tumor volume. PET/CT: positron emission tomography/computer tomography. SCC: Squamous cell carcinoma. AC: Adenocarcinoma.
