# Supplementary material for: Circulating cell free DNA during definitive chemo-radiotherapy in non-small cell lung cancer patients – initial observations
Source: PLoS One. 2020 Apr 28;15(4):e0231884. doi: 10.1371/journal.pone.0231884 (PMC7188247; doi:10.1371/journal.pone.0231884)
Supplement: Attachment — Submitted filename: Response to reviewers.docx [file pone.0231884.s005.docx]

PONE-D-19-24047
Circulating cell free DNA during definitive chemo-radiotherapy in non-small cell lung cancer patients – initial observations
PLOS ONE

Dear Dr. Nygård,

Thank you for submitting your manuscript to PLOS ONE. After careful consideration, we feel that it has merit but does not fully meet PLOS ONE’s publication criteria as it currently stands. Therefore, we invite you to submit a revised version of the manuscript that addresses the points raised during the review process.

Please address the single reviewer's comments.

If you would like to make changes to your financial disclosure, please include your updated statement in your cover letter.

To enhance the reproducibility of your results, we recommend that if applicable you deposit your laboratory protocols in [protocols.io](https://eur03.safelinks.protection.outlook.com/?url=http%3A%2F%2Fprotocols.io&data=01%7C01%7CMalene.fischer%40kcl.ac.uk%7Ca843914cac224eba421808d794fb1232%7C8370cf1416f34c16b83c724071654356%7C0&sdata=FjaO8HDQsLEGbmHhMfggyAI5Yfb5MWmkxQCZf%2FSQxD8%3D&reserved=0), where a protocol can be assigned its own identifier (DOI) such that it can be cited independently in the future. For instructions see: [http://journals.plos.org/plosone/s/submission-guidelines#loc-laboratory-protocols](https://eur03.safelinks.protection.outlook.com/?url=http%3A%2F%2Fjournals.plos.org%2Fplosone%2Fs%2Fsubmission-guidelines%23loc-laboratory-protocols&data=01%7C01%7CMalene.fischer%40kcl.ac.uk%7Ca843914cac224eba421808d794fb1232%7C8370cf1416f34c16b83c724071654356%7C0&sdata=vZVsFdIJUcdA7LuafkpAHFnHm9ZV5YHq6y%2FBlyiYxIU%3D&reserved=0)

Please include the following items when submitting your revised manuscript:

- A rebuttal letter that responds to each point raised by the academic editor and reviewer(s). This letter should be uploaded as separate file and labeled 'Response to Reviewers'.
- A marked-up copy of your manuscript that highlights changes made to the original version. This file should be uploaded as separate file and labeled 'Revised Manuscript with Track Changes'.
- An unmarked version of your revised paper without tracked changes. This file should be uploaded as separate file and labeled 'Manuscript'.

Please note while forming your response, if your article is accepted, you may have the opportunity to make the peer review history publicly available. The record will include editor decision letters (with reviews) and your responses to reviewer comments. If eligible, we will contact you to opt in or out.

We look forward to receiving your revised manuscript.

Kind regards,

Jeffrey Chalmers, Ph.D.
Academic Editor
PLOS ONE

Journal Requirements:

When submitting your revision, we need you to address these additional requirements.

1. Please ensure that your manuscript meets PLOS ONE's style requirements, including those for file naming. The PLOS ONE style templates can be found at

[http://www.journals.plos.org/plosone/s/file?id=wjVg/PLOSOne_formatting_sample_main_body.pdf](https://eur03.safelinks.protection.outlook.com/?url=http%3A%2F%2Fwww.journals.plos.org%2Fplosone%2Fs%2Ffile%3Fid%3DwjVg%2FPLOSOne_formatting_sample_main_body.pdf&data=01%7C01%7CMalene.fischer%40kcl.ac.uk%7Ca843914cac224eba421808d794fb1232%7C8370cf1416f34c16b83c724071654356%7C0&sdata=whKzJSmJb%2B3%2BOEYYhroRLeB7GoVdhLrqO%2BeYuXw51%2F8%3D&reserved=0) and [http://www.journals.plos.org/plosone/s/file?id=ba62/PLOSOne_formatting_sample_title_authors_affiliations.pdf](https://eur03.safelinks.protection.outlook.com/?url=http%3A%2F%2Fwww.journals.plos.org%2Fplosone%2Fs%2Ffile%3Fid%3Dba62%2FPLOSOne_formatting_sample_title_authors_affiliations.pdf&data=01%7C01%7CMalene.fischer%40kcl.ac.uk%7Ca843914cac224eba421808d794fb1232%7C8370cf1416f34c16b83c724071654356%7C0&sdata=RwsCgUr2OZ9olBNUPZnEPezX4FwY8Y%2FFbFTFq5PT6SI%3D&reserved=0)

2. In your Methods section, please provide additional information about the participant recruitment method. Specifically, please provide the dates of enrolment (month/year) and a description of how the participants were recruited.

This has been added in the methods section as required; (page 5)

…inclusion “at their first visit to the Department of Oncology before starting chemotherapy. Patients were offered 24 hours of consideration and could give their acceptance just before first cycle of chemotherapy. Patients were enrolled in the study from October 2016 until September 2017 at the Department of Oncology, University Hospital of Copenhagen, Rigshospitalet, Denmark.”

3. Your ethics statement must appear in the Methods section of your manuscript. If your ethics statement is written in any section besides the Methods, please move it to the Methods section and delete it from any other section. Please also ensure that your ethics statement is included in your manuscript, as the ethics section of your online submission will not be published alongside your manuscript.

The ethics statement appears in the Methods section, ( page 5)

“All patients signed an informed consent prior to inclusion. The study was approved by the Danish Ethics Committee, protocol number H-16029201 and the Danish Data Protection Agency, 2012-58-0004/RH-2016-237/ I-suite no 04837 and by the institutional review board.”

4. Please include captions for your Supporting Information files at the end of your manuscript, and update any in-text citations to match accordingly. Please see our Supporting Information guidelines for more information: [http://journals.plos.org/plosone/s/supporting-information](https://eur03.safelinks.protection.outlook.com/?url=http%3A%2F%2Fjournals.plos.org%2Fplosone%2Fs%2Fsupporting-information&data=01%7C01%7CMalene.fischer%40kcl.ac.uk%7Ca843914cac224eba421808d794fb1232%7C8370cf1416f34c16b83c724071654356%7C0&sdata=zLJLBrpIi51Rjv1B2qtwDCiwqP%2FPsxe8D2UbOTPymsA%3D&reserved=0)

[Note: HTML markup is below. Please do not edit.]

Reviewers' comments:

Reviewer's Responses to Questions

**Comments to the Author**

1. Is the manuscript technically sound, and do the data support the conclusions?

The manuscript must describe a technically sound piece of scientific research with data that supports the conclusions. Experiments must have been conducted rigorously, with appropriate controls, replication, and sample sizes. The conclusions must be drawn appropriately based on the data presented.

Reviewer #1: Yes

2. Has the statistical analysis been performed appropriately and rigorously?

Reviewer #1: Yes

3. Have the authors made all data underlying the findings in their manuscript fully available?

The [PLOS Data policy](https://eur03.safelinks.protection.outlook.com/?url=http%3A%2F%2Fwww.plosone.org%2Fstatic%2Fpolicies.action%23sharing&data=01%7C01%7CMalene.fischer%40kcl.ac.uk%7Ca843914cac224eba421808d794fb1232%7C8370cf1416f34c16b83c724071654356%7C0&sdata=IW229fmZvDUXx4R%2FARE8hFv6t1W2ozoCj3pO%2Bj8DftE%3D&reserved=0) requires authors to make all data underlying the findings described in their manuscript fully available without restriction, with rare exception (please refer to the Data Availability Statement in the manuscript PDF file). The data should be provided as part of the manuscript or its supporting information, or deposited to a public repository. For example, in addition to summary statistics, the data points behind means, medians and variance measures should be available. If there are restrictions on publicly sharing data—e.g. participant privacy or use of data from a third party—those must be specified.

Reviewer #1: Yes

4. Is the manuscript presented in an intelligible fashion and written in standard English?

PLOS ONE does not copyedit accepted manuscripts, so the language in submitted articles must be clear, correct, and unambiguous. Any typographical or grammatical errors should be corrected at revision, so please note any specific errors here.

Reviewer #1: Yes

5. Review Comments to the Author

Please use the space provided to explain your answers to the questions above. You may also include additional comments for the author, including concerns about dual publication, research ethics, or publication ethics. (Please upload your review as an attachment if it exceeds 20,000 characters)

Reviewer #1: • The data demonstrate that there was no concordance between radiological response and cfDNA change during treatment. This in line with previous observations which have shown a lack of predictive accuracy for total cfDNA (Weiss G et al, Clin Cancer Res 2017; 23: 5074-81). The paper of Weiss, however, demonstrated that on the other hand chromosomal instability quantification in plasma cfDNA was associated with therapeutic response to immune therapy. This paper should be mentioned in the publication.

Thank you for this very valid comment. The paper of Weiss G et al is very relevant in this manuscript, so thank you. It is now cited as ref 13 (page 4)

• P. 6: The authors state that samples were analyzed “… using sWGS, resembling the method of plasma-Seq” and give a citation. Some more detailed information on how they applied this method should perhaps be given in the supplement.

Further details have been added in the manuscript: (page 7+8, line 150-170):

To confirm cfDNA counts to contain DNA from tumor cells, part two blood samples were analyzed using sWGS assessing somatic copy number alterations (SCNAs) in a genome-wide manner. Genomic alterations including SCNAs are a key characteristic of the cancer genome ^15^ and the detection of SCNAs in cfDNA can thus indicate the presence of tumor DNA in the circulation. The principle of sWGS relies on low-depth (>0.1x) whole-genome sequencing to estimate the SCNAs from the sequencing read depth ^16, 17^. Indexed DNA libraries were constructed from 10 ng of cfDNA using the NEBNext Ultra II DNA library Prep Kit for Illumina (New England Biolabs) and paired-end sequenced (150-bp) on the MiSeq or NextSeq platforms (Illumina). Prior to sequencing, the libraries were quantified using the high sensitivity dsDNA assay on the Qubit instrument (Thermo Fisher Scientific) and the fragment size inspected using the Bioanalyzer High Sensitivity DNA Analysis on the 2100 Bioanalyzer Instrument (Agilent). Furthermore, the libraries were pooled in equimolar amounts with six libraries in each run. A minimum of 5 million reads per sample were required to evaluate copy number alterations in cfDNA. Analysis of sWGS data was performed using the QDNASeq (<https://bioconductor.org/packages/release/bioc/html/QDNAseq.html>) dividing the genome into windows or bins of a certain size, and the GC-corrected read count for each bin is then used to determine if the region is increased or decreased.

Due to the low content of tumor cfDNA in this group of patients, an *in silico* size selection algorithm selecting fragments between 90 and 150 bp was applied to increase the detection of SCNAs ^17^. This metric is called trimmed median absolute deviation from copy number neutrality (tMAD) and has been described by Mouliere and collegues ^17^.

• To what extent was the sensitivity of somatic copy number alterations detection increased?

tMAD is a conversion of the SCNA signal, by nature qualitative, in a quantitative metric, that could then be compared to other ctDNA metrics (like VAF, tumor fraction, …).  Figure 4B of the STM paper (ref 17) indicate the correlation score between MAF (from sequencing) and tMAD (from sWGS). With size selection (Figure 4D) AUC =0.9. Without size selection AUC 0.69.

The ROC curve in figure 4D indicate the increased in AUC after in silico size selection using tMAD metrics on a large cohort of cases.

In this present study, we found that processing sWGS data by size selection with tMAD > 0.015, SCNAs were detected in 16 out of 23 samples (70%) compared to 5 samples (22%) without size selection (Fig 5).

Please note:

We found an error in the calculations (16 out of 23 and not 17 out of 23 samples). The percentage is corrected to 70% and not 74%. This has been corrected in the manuscript: page 12 :

“Noticeably, processing sWGS data by size selection with tMAD > 0.015, SCNAs were detected in 16 out of 23 samples (70%) compared to 5 samples (22%) without size selection (Fig 5).”

• P. 9: The authors state that the mean increase in cfDNA was different in AC and SCC patients. Was this difference statistically significant?

We ran an unpaired Wilcoxon rank sum test. P-value 0.7. This is added in the manuscript (page 10)

• P. 11: What was the detectable ctDNA fraction with their method? In this context, the authors could cite a paper of Fiala et al (J Appl Lab Med 2018; 03: 300-313) which provides information on the likelihood of ctDNA detection related to detectable fraction.

Thank you for your comment.

In this study we did not directly investigate a specific genomic alteration since we did not have the appropriate tumor tissue for genomic profiling.

We have cited Fiala et al in the manuscript (page 12) including the STM paper from Mouliere and colleagues (ref 17) to highlight the likelihood of ctDNA detection.

The following sentence has been added to the manuscript.

“Using size selection and tMAD scores have shown ctDNA detection down to a minor allele frequency of 0.01 (1%).”

6. PLOS authors have the option to publish the peer review history of their article ([what does this mean?](https://eur03.safelinks.protection.outlook.com/?url=https%3A%2F%2Fjournals.plos.org%2Fplosone%2Fs%2Feditorial-and-peer-review-process%23loc-peer-review-history&data=01%7C01%7CMalene.fischer%40kcl.ac.uk%7Ca843914cac224eba421808d794fb1232%7C8370cf1416f34c16b83c724071654356%7C0&sdata=khaOTsITfPzC40QmrZqP1Ji%2ByDkkseaZ6bLFhvlo%2BW8%3D&reserved=0)). If published, this will include your full peer review and any attached files.


If you choose “no”, your identity will remain anonymous but your review may still be made public.


**Do you want your identity to be public for this peer review?** For information about this choice, including consent withdrawal, please see our [Privacy Policy](https://eur03.safelinks.protection.outlook.com/?url=https%3A%2F%2Fwww.plos.org%2Fprivacy-policy&data=01%7C01%7CMalene.fischer%40kcl.ac.uk%7Ca843914cac224eba421808d794fb1232%7C8370cf1416f34c16b83c724071654356%7C0&sdata=ZzlsswiFhZB1ix4L3JH%2FSEsnmlghng7eAKEY%2FZ8PXis%3D&reserved=0).

Reviewer #1: Yes: Michael Oellerich


[NOTE: If reviewer comments were submitted as an attachment file, they will be attached to this email and accessible via the submission site. Please log into your account, locate the manuscript record, and check for the action link "View Attachments". If this link does not appear, there are no attachment files to be viewed.]


While revising your submission, please upload your figure files to the Preflight Analysis and Conversion Engine (PACE) digital diagnostic tool, [https://pacev2.apexcovantage.com/](https://eur03.safelinks.protection.outlook.com/?url=https%3A%2F%2Fpacev2.apexcovantage.com%2F&data=01%7C01%7CMalene.fischer%40kcl.ac.uk%7Ca843914cac224eba421808d794fb1232%7C8370cf1416f34c16b83c724071654356%7C0&sdata=LUIZCpWMFbnhQ3P26WJmkSJovLP4P%2Byrg7HXZi2NwOg%3D&reserved=0). PACE helps ensure that figures meet PLOS requirements. To use PACE, you must first register as a user. Registration is free. Then, login and navigate to the UPLOAD tab, where you will find detailed instructions on how to use the tool. If you encounter any issues or have any questions when using PACE, please email us at [figures@plos.org](mailto:figures@plos.org). Please note that Supporting Information files do not need this step.
